# XGBoost Outperforms Traditional Transplant Regulatory Models Based on Regression

**DOI:** 10.1097/AS9.0000000000000698

**Published:** 2026-08-05

**Authors:** Rowland W. Pettit, Nicholas J. Cione, Britton B. Marlatt, Selim Uzgoren, Anil P. Shetty, Gwendolyn Henry, Jim Havelka, John A. Goss, Abbas A. Rana

**Affiliations:** From the * Department of Pathology, Mass General Brigham, Harvard Medical School, Boston, MA; † Department of Student Affairs, Baylor College of Medicine, Houston, TX; ‡ Research and Development, InformAI, Houston, TX; § Division of Abdominal Transplantation, Department of Surgery, Baylor College of Medicine, Houston, TX.

**Keywords:** artificial intelligence, graft survival, machine learning, outcome prediction, patient survival, transplant

## Abstract

**Objective:**

To determine whether machine learning (ML) models, specifically Extreme Gradient Boosting (XGBoost), improve the prediction of graft and patient survival compared with Scientific Registry of Transplant Recipients (SRTR) regression models across heart, lung, liver, and kidney transplants.

**Background:**

Accurate prediction of post-transplant outcomes is essential for organ allocation and regulatory oversight. Current SRTR models rely on regression techniques that may not capture complex donor-recipient interactions. ML offers the potential for improved predictive accuracy.

**Methods:**

A retrospective cohort study analyzed United Network for Organ Sharing data from 1987 to 2023 for heart, lung, liver, and kidney transplants. Cox proportional hazards models (SRTR) were reconstructed and compared with XGBoost models. Outcomes included graft and patient survival at 1 and 3 years. Model performance was assessed using area under the curve and DeLong’s test.

**Results:**

XGBoost models outperformed SRTR models for 1-year graft survival: heart (area under the curve 0.698 *vs* 0.576), kidney (0.736 *vs* 0.649), liver (0.706 *vs* 0.616), and lung (0.612 *vs* 0.572). Similar improvements were observed for patient survival and 3-year outcomes, with statistical significance for most organs except lung graft survival at 1 year.

**Conclusions and Relevance:**

ML models provide superior predictive accuracy compared with traditional regression-based models for transplant outcomes. While current clinical utility remains limited, these findings support further development and integration of ML techniques to enhance regulatory evaluations and optimize patient care.

## INTRODUCTION

The field of solid organ transplantation faces a persistent and severe shortage of donor allografts. In 2022, over 180,000 patients in the United States awaited transplants, yet only 46,629 transplants were performed.^1^ In this environment, regulation is critical to optimize patient outcomes and ensure equitable allocation of allografts. Against this backdrop, transplantation has evolved into a highly regulated specialty, relying predominantly on regression-based models to predict risk, evaluate transplant programs, and ensure acceptable results.^2^

Currently, the regulatory landscape is anchored by traditional linear and logistic regression models. The Organ Procurement and Transplantation Network, under the administration of the United Network for Organ Sharing (UNOS), maintains a vast national transplant database. The Scientific Registry of Transplant Recipients (SRTR) leverages these data to build regression-based models that predict graft and patient survival.^2^ The Health Resources and Services Administration relies on these models to evaluate program performance, while insurance companies use these models to determine centers of excellence. Researchers have developed additional risk scores to assess risk, prognosticate, and avoid futile transplants. The model for end-stage liver disease (MELD) estimates waitlist mortality to prioritize patient need and was adopted into policy by UNOS in 2002.^3^ Post-transplant scores such as the Survival Outcome Following Liver Transplantation (SOFT), Balance of Risk (BAR), and Donor Risk Index function to predict surgical outcomes.^4,5^ Similarly, the adoption of the Lung Allocation Score (LAS) improved both waitlist mortality and 1-year post-transplant survival in lung recipients,^6,7^ and the Index for Mortality Prediction After Cardiac Transplantation (IMPACT) score aids in estimating short-term heart transplant survival.^8^ For kidney transplantation, the iBox system offers promising accuracy in predicting graft failure but lacks broad validation in US data.^9^ While these models have contributed valuable insights, their utility is limited by their underlying linear assumptions, which fail to fully capture the complex, nonlinear interactions between donor and recipient characteristics.

Artificial intelligence (AI) and machine learning (ML) methods offer improved post-transplant outcome prediction by capturing nonlinear donor-recipient interactions that conventional regression cannot model. ML has been applied to transplant outcomes across kidney,^10^ liver,^11^ lung,^12^ and heart transplants.^13^ Several studies using UNOS data are particularly relevant to the present work. Kantidakis et al^14^ compared custom-derived Cox models to neural networks for post-transplant survival on UNOS data (n = 62,294, 97 features included) and found that ML did not substantially outperform Cox regression. Similarly, Guijo-Rubio et al^15^ applied gradient boosting to UNOS liver data (n = 39,095, 29 features included) across survival horizons of up to 9 years, with only modest gains over Cox models. Ershoff et al^16^ applied a deep neural network to UNOS data (n = 57,544, 202 features included) to predict 3-month post-transplant mortality with limited discriminative improvement over regression. Nitski et al^17^ demonstrated stronger performance in liver transplant using a transformer architecture but critically relied on longitudinal post-transplant laboratory data accumulated over time–a fundamentally different task than predicting outcomes at the time of organ allocation. Across this body of work, no study has directly compared ML against the SRTR regulatory Cox models on the same UNOS cohort those models are built on.

Existing regulatory models often have limited predictive value, creating an opportunity for novel methods to improve their accuracy. Previously mentioned ML models trained on the Organ Procurement and Transplantation Network database show promise to enhance predictions of post-transplant outcomes. More accurate predictions will provide fairer regulation of centers, help to optimize outcomes, and potentially avoid futile transplants. This study aims to develop and evaluate predictive models for post-transplant outcomes using ML algorithms and directly compare them against the existing SRTR regression-based models.

## METHODS

### Study Design and Data Sources

We conducted a retrospective cohort study to develop and evaluate predictive models for post-transplant outcomes across 4 organ types: heart, lung, liver, and kidney. De-identified datasets were obtained from the UNOS, encompassing donor and recipient information for transplants performed between 1987 and June 2023. These datasets included donor demographics, medical history, laboratory values, and organ-specific information, as well as recipient demographics, medical history, transplant details, and follow-up outcomes. Additionally, waitlist data provided information on candidates awaiting transplantation, including wait times and outcomes.

### Data Cleaning and Standardization

To ensure consistency and reliability of the data, we standardized variable names and labels by converting them to lowercase and replacing spaces and special characters with underscores. Duplicate entries and inconsistent records were identified and removed to maintain data integrity. Missing values in key variables were addressed by creating binary missing indicators, aligning with methodologies used by the SRTR.

### Merging Datasets

For each organ type, donor and recipient datasets were merged using unique identifiers to create comprehensive datasets containing both donor and recipient information. Specifically, heart and lung transplant datasets were merged on the unique identifier for thoracic data; liver transplant datasets were merged on the liver data unique identifier; and kidney transplant datasets were merged on the kidney-pancreas data unique identifier.

### Outcome Variable Creation

We generated survival outcome variables for graft survival and patient survival at multiple time points: 1 month, 3 months, 1 year, 3 years, 5 years, and 10 years post-transplant. Graft survival was defined as the time from transplant to graft failure or last recorded follow-up, and patient survival was defined as the time from transplant to patient death or last recorded follow-up. Cases with zero survival time were excluded to focus on post-transplant outcomes.

### Feature Engineering

To enhance predictive power, we meticulously recreated variables used in the publicly available SRTR risk models. UNOS variables were mapped to SRTR variables based on definitions provided in SRTR technical documentation. Variables were transformed to match SRTR specifications, including categorization and scaling. Missing data indicators were created by generating binary variables for missing values in critical variables. Additional feature construction included calculating donor-recipient ratios, such as age and body mass index (BMI) ratios, and time-based calculations like wait times, ischemic times, and time on dialysis for kidney transplants. Categorical variables, such as education level and diabetes status, were encoded appropriately. Human leukocyte antigen mismatch levels were calculated to assess immunological compatibility. Organ-specific scores were also computed: the MELD scores for liver transplants and panel reactive antibody levels for kidney transplants.

### Model Development

We developed 2 types of models for each organ and outcome: Cox proportional hazards models and Extreme Gradient Boosting (XGBoost) models. The SRTR Cox proportional hazards models were reconstructed using the exact variable coefficients and interaction terms as published by SRTR. Models were stratified by transplant year to account for temporal trends, and baseline hazard functions were estimated using the Breslow method. XGBoost models were developed as an advanced ML alternative. Datasets were divided into training (70%), validation (15%), and test (15%) sets, stratified by outcome. Hyperparameters such as learning rate, maximum tree depth, and regularization parameters were tuned using Bayesian optimization. Five-fold cross-validation was employed within the training set to prevent overfitting. Feature importance was analyzed to interpret the contribution of each variable to the model’s predictions.

### Model Evaluation

Probability predictions were generated for each outcome and time point. For the Cox models, survival probabilities were calculated using the baseline hazard function and individual risk scores. For the XGBoost models, predicted probabilities were obtained directly from the model outputs. Receiver operating characteristic (ROC) curves were constructed, and the area under the curve (AUC) was calculated to evaluate model performance at 1 month, 3 months, 1 year, 3 years, 5 years, and 10 years post-transplant. The AUCs of the SRTR and XGBoost models were compared using DeLong’s test to assess statistical significance. Calibration plots and Brier scores were used to assess the agreement between predicted probabilities and observed outcomes.

### Statistical Analysis

For statistical analysis, DeLong’s test was employed to compare the AUCs of the SRTR and XGBoost models. The null hypothesis was that there was no difference between the two ROC curves, with a p-value less than 0.05 considered statistically significant. Models were also evaluated within specific subgroups, including transplant era (transplants performed between 2017–2019 and 2019–2022), organ type (heart, lung, liver, and kidney transplants), and outcome type (graft survival and patient survival). All analyses were conducted using R version 4.0.5. Key packages included data.table, dplyr, and tidyr for data manipulation; survival, xgboost, and pROC for statistical modeling; ParBayesianOptimization for hyperparameter optimization; and ggplot2, knitr, and tableone for visualization. As the study utilized de-identified data from UNOS, ethical approval and patient consent were not required. All data handling complied with relevant data protection and privacy regulations.

### Supplemental Methods

Further details on data preprocessing, model architecture, and hyperparameter optimization are available in Supplemental Methods, https://links.lww.com/AOSO/A653.

## RESULTS

### Overall Model Performance

The performance of the models was assessed by comparing the SRTR Cox proportional hazards models and the XGBoost ML models across all organs for both graft and patient survival at 1-year and 3-year time points. ROC curves were generated, and the AUC values were calculated to evaluate predictive accuracy. XGBoost models consistently demonstrated higher AUC values compared with SRTR models, indicating superior predictive performance.

### Demographics

Demographics before cleaning data may be found in Supplemental Table 1, https://links.lww.com/AOSO/A653.

### One-Year Graft Survival

**FIGURE 1.**
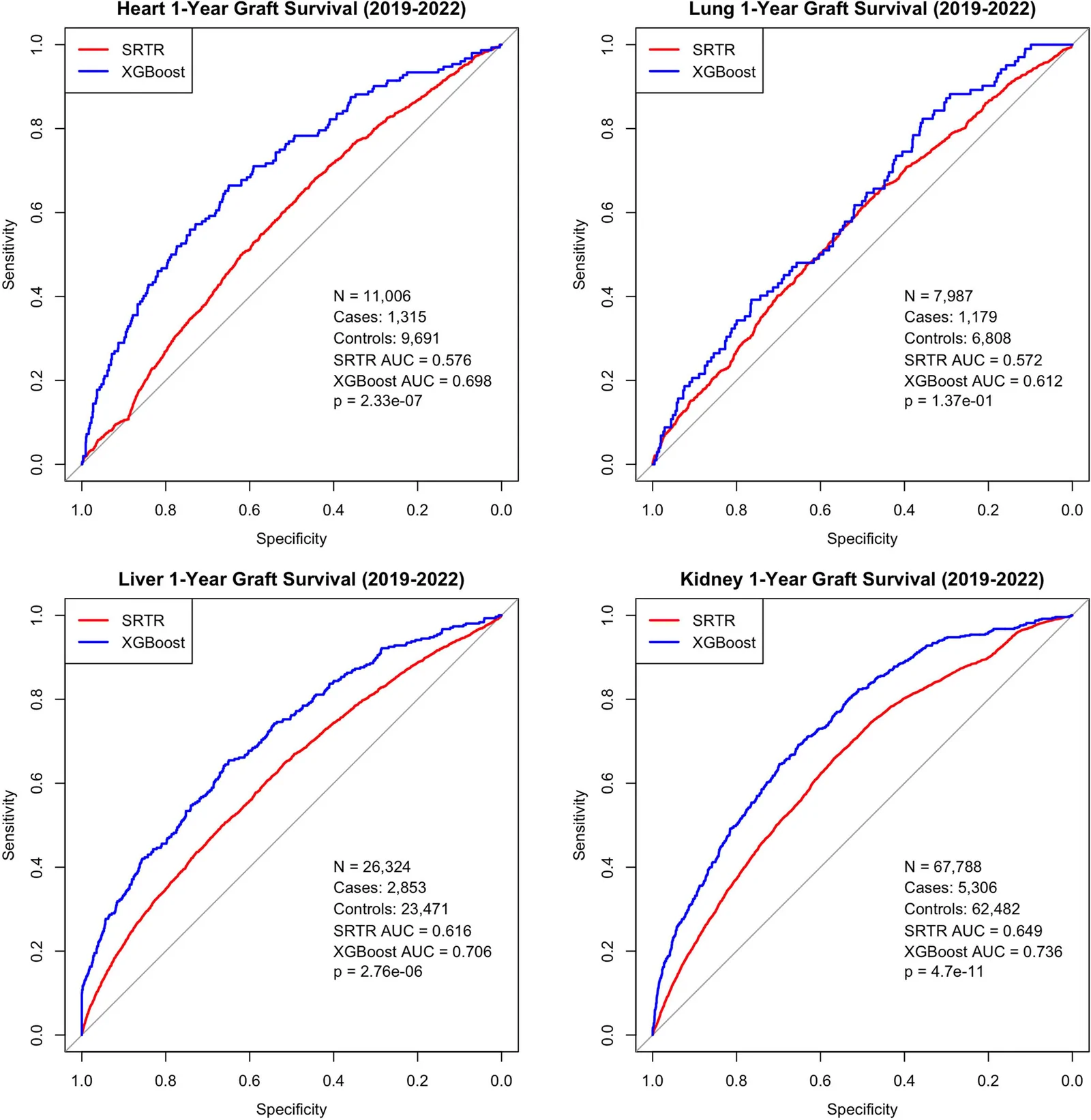
ROC curves comparing SRTR and XGBoost models for 1-year graft survival across organs: (A) Heart, (B) Lung, (C) Liver, (D) Kidney.

For 1-year graft survival predictions, XGBoost models outperformed SRTR models across all four organs (Fig. 1). In heart transplants, the XGBoost model achieved an AUC of 0.698 compared with 0.576 for the SRTR model, a significant improvement (*P* = 2.33 × 10^–7^). Kidney transplants showed a similar trend, with the XGBoost model attaining an AUC of 0.736 *versus* 0.649 for the SRTR model (*P* = 4.70 × 10^–11^). Liver transplants also benefited from the ML approach, with the XGBoost model achieving an AUC of 0.706 compared with 0.616 for the SRTR model (*P* = 2.76 × 10^–6^). In lung transplants, the XGBoost model’s AUC improved to 0.612 from the SRTR model’s 0.572; however, this improvement was not statistically significant (*P* = 0.137).

### One-Year Patient Survival

**FIGURE 2.**
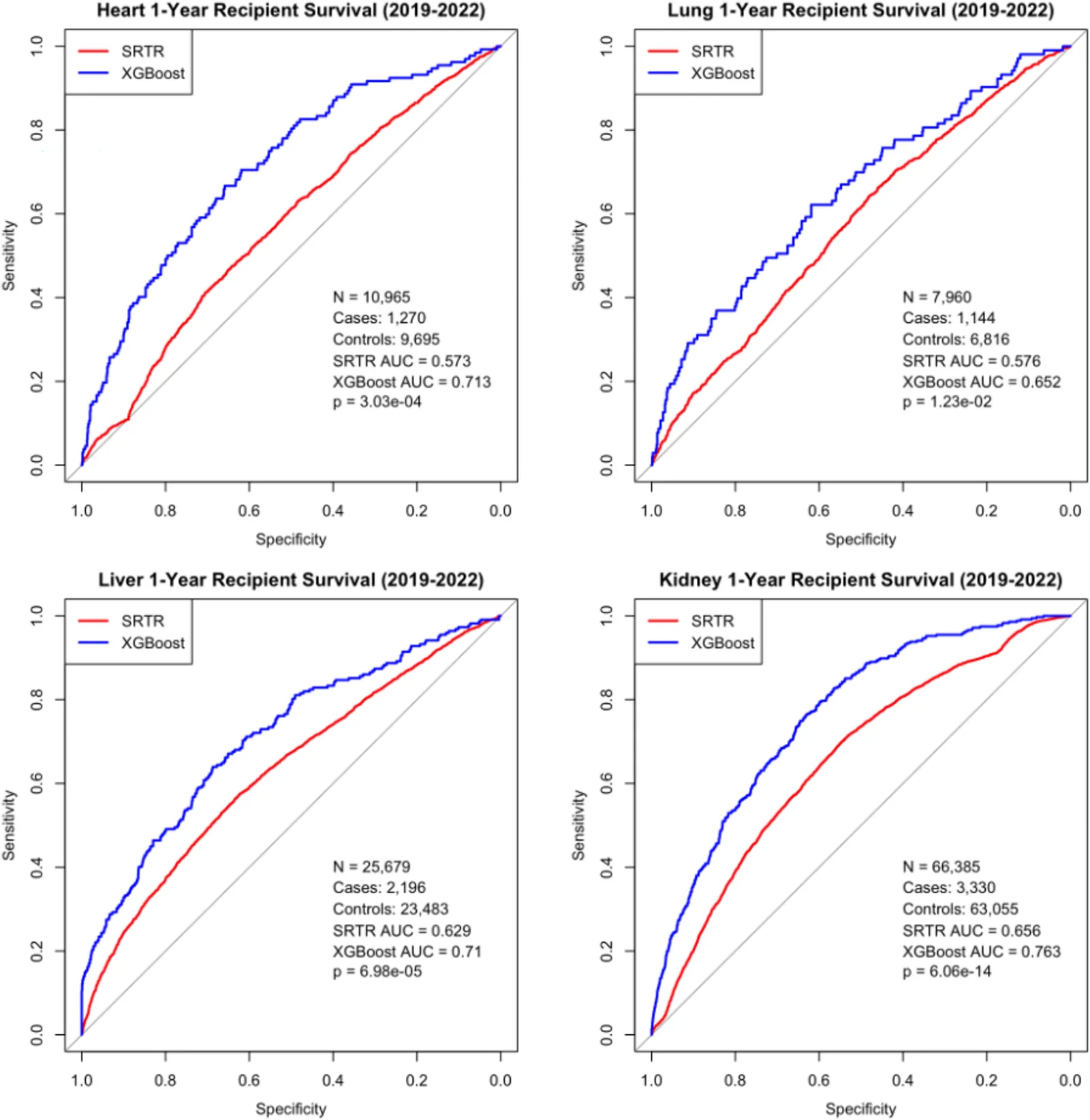
ROC curves comparing SRTR and XGBoost models for 1-year patient survival across organs: (A) Heart, (B) Lung, (C) Liver, (D) Kidney.

Similar enhancements were observed in 1-year patient survival predictions (Fig. 2). The XGBoost model for heart transplants achieved an AUC of 0.713 compared with 0.573 for the SRTR model (*P* = 3.03 × 10^–4^). In kidney transplants, the XGBoost model significantly outperformed the SRTR model with an AUC of 0.763 *versus* 0.656 (*P* = 6.06 × 10^–14^). Liver transplants saw an improvement with the XGBoost model achieving an AUC of 0.710 compared with 0.629 for the SRTR model (*P* = 6.98 × 10^–5^). Lung transplants also showed a statistically significant improvement, with the XGBoost model reaching an AUC of 0.652 *versus* 0.576 for the SRTR model (*P* = 1.23 × 10^–2^).

### Three-Year Graft Survival

**FIGURE 3.**
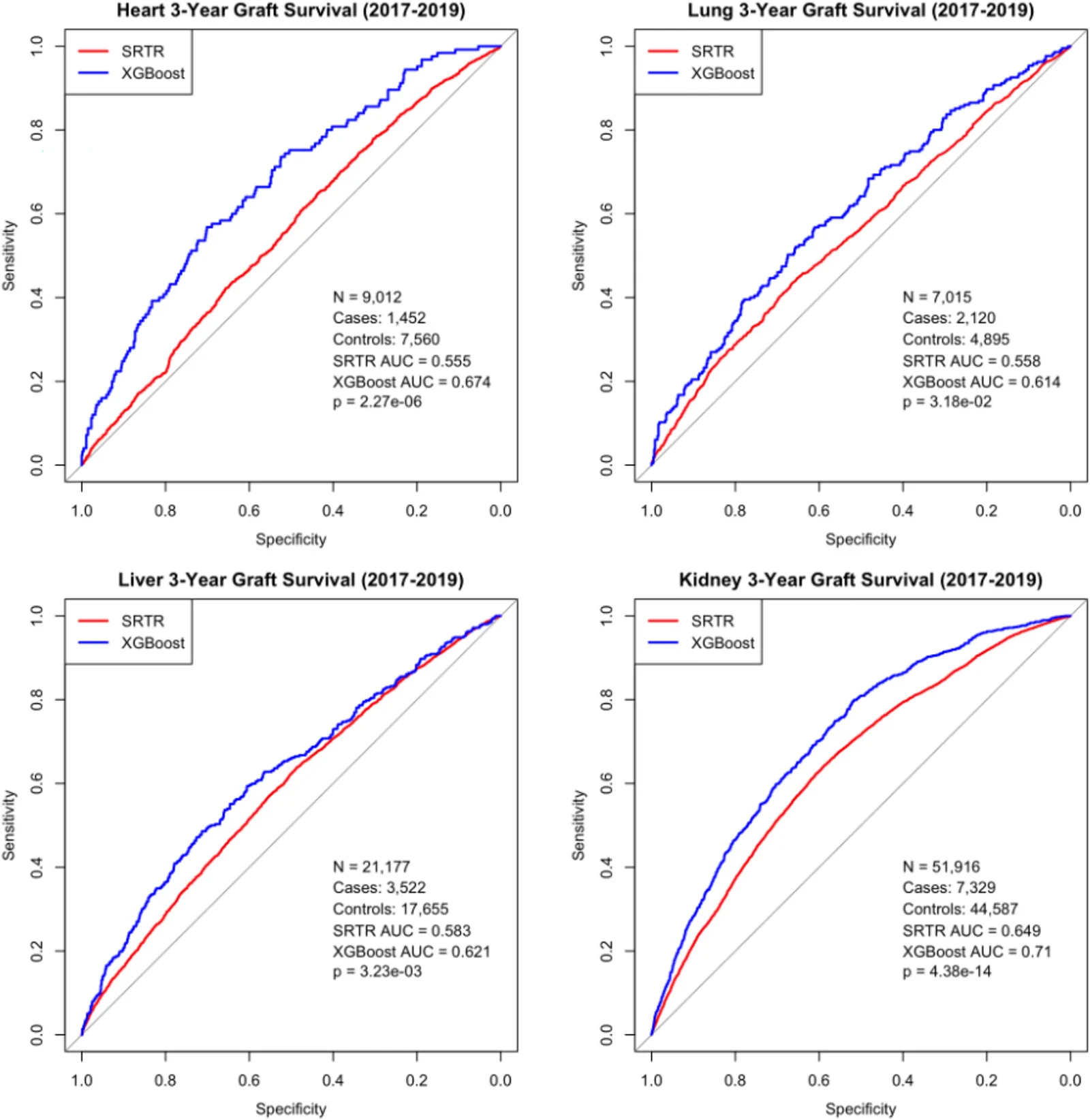
ROC curves comparing SRTR and XGBoost models for 3-year graft survival across organs: (A) Heart, (B) Lung, (C) Liver, (D) Kidney.

For 3-year graft survival predictions, XGBoost models continued to outperform SRTR models across all organs (Fig. 3). In heart transplants, the XGBoost model achieved an AUC of 0.674 compared with 0.555 for the SRTR model (*P* = 2.27 × 10^–6^). The kidney transplant XGBoost model had an AUC of 0.710 *versus* 0.649 for the SRTR model (*P* = 4.38 × 10^–14^). Liver transplants showed an improvement, with the XGBoost model achieving an AUC of 0.621 compared with 0.583 for the SRTR model (*P* = 3.23 × 10^–3^). Lung transplants had a modest but statistically significant enhancement, with the XGBoost model’s AUC increasing to 0.614 from 0.558 for the SRTR model (*P* = 3.18 × 10^–2^).

### Three-Year Patient Survival

**FIGURE 4.**
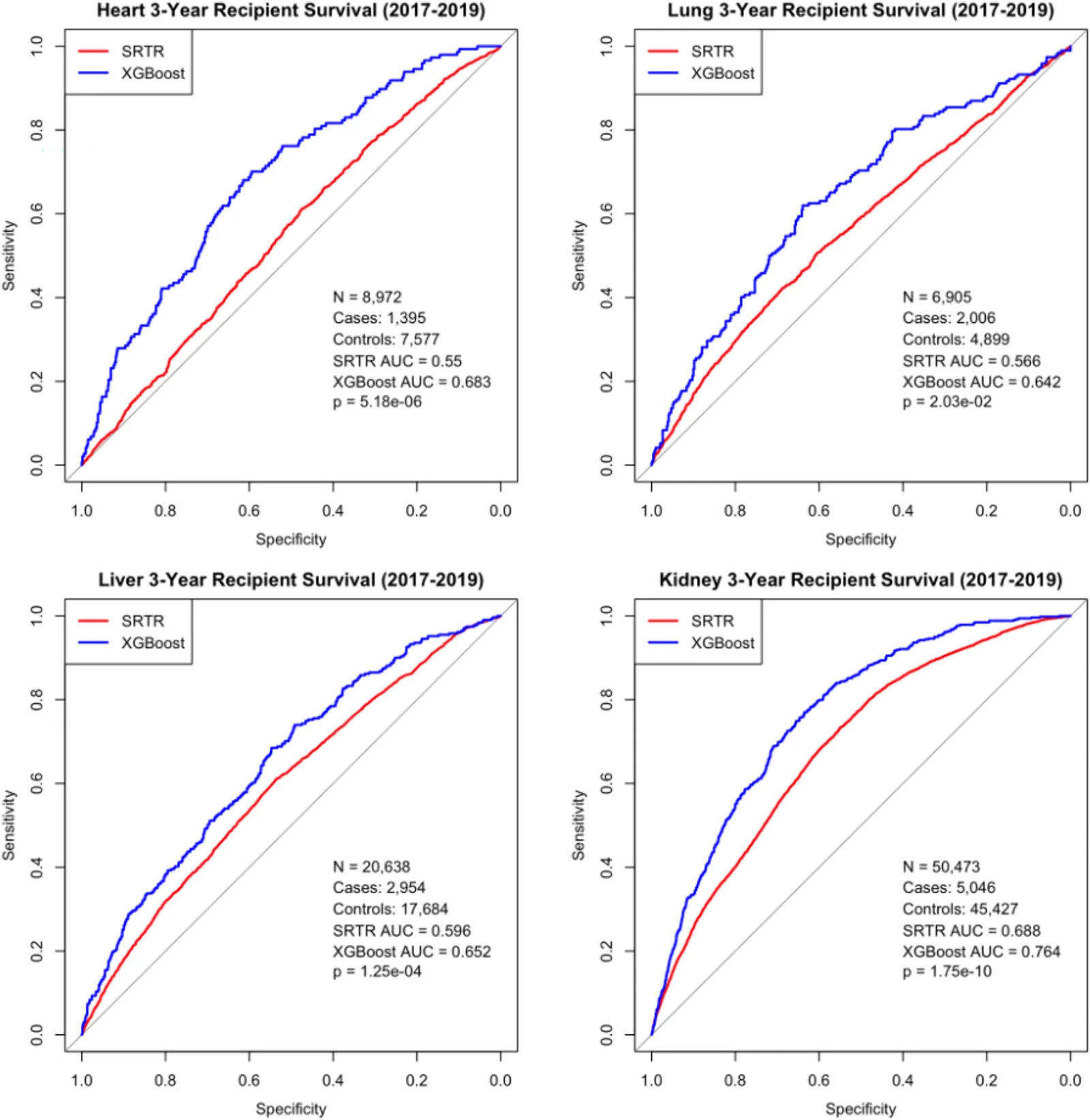
ROC curves comparing SRTR and XGBoost models for 3-year patient survival across organs: (A) Heart, (B) Lung, (C) Liver, (D) Kidney.

In 3-year patient survival predictions, XGBoost models again demonstrated superior performance (Fig. 4). The heart transplant XGBoost model achieved an AUC of 0.683 compared with 0.550 for the SRTR model (*P* = 5.18 × 10^–6^). Kidney transplants showed significant improvement, with the XGBoost model’s AUC at 0.764 *versus* 0.688 for the SRTR model (*P* = 1.75 × 10^–10^). For liver transplants, the XGBoost model achieved an AUC of 0.652 compared with 0.596 for the SRTR model (*P* = 1.25 × 10^–4^). Lung transplants also benefited, with the XGBoost model’s AUC increasing to 0.642 from 0.566 for the SRTR model (*P* = 2.03 × 10^–2^).

### Statistical Comparison and Sample Sizes

Supplemental Table 2, https://links.lww.com/AOSO/A653 summarizes the AUC values, Brier scores, sample sizes, number of cases and controls, and *P*-values from DeLong’s test comparing SRTR and XGBoost models across organ types and outcomes. In all comparisons, XGBoost models demonstrated higher AUC values, and the differences were statistically significant (*P* < 0.05) in most cases. The Brier scores were improved in all XGBoost models, except for 1-year and 3-year kidney patient survival models.

### Summary of Results

Overall, the XGBoost ML models demonstrated superior predictive performance over traditional SRTR regression models across all organ types and outcomes. Significant improvements in AUC values were observed, particularly in heart and kidney transplants. While the improvements in lung transplants were less pronounced, they were still statistically significant in most cases. The enhanced predictive accuracy of XGBoost models suggests that algorithms enhanced by ML can provide more precise risk stratification for post-transplant outcomes, potentially leading to better patient management and allocation of resources.

## DISCUSSION

This study demonstrates accurate ML models that outperform current SRTR regression-based regulatory models. More accurate regulatory models will enhance evaluations of transplant centers, improve survival outcomes, and strengthen patient decision-making. ML achieves this performance through its ability to capture complex relationships between donor and recipient variables that would otherwise go undetected by current regression methods. Across the 4 most common transplanted organs—kidney, liver, heart, and lung—XGBoost consistently achieves higher AUC values than the regression-based models currently employed by SRTR. Heart transplant models derived the greatest benefit from ML, with an average improvement of 0.128 (22.81%) over SRTR models. Other improvements include a 12.5% average improvement for kidney models and a 10.9% average improvement for lung and liver models when compared with the existing SRTR counterparts. Notably, all kidney outcome models surpassed an AUC of 0.7, often held as a threshold for clinical utility. Though the results may not represent significant improvement in clinical utility, they provide a proof-of-concept to build more clinically relevant models in the future. ML techniques will undoubtedly improve over time and will likely significantly outperform regression in the future, thereby offering more sound and equitable regulatory oversight.

The implications of these findings are important for the broader field of transplantation, which is characterized by a profound shortage of donor allografts and intensive regulatory scrutiny. UNOS provides a national registry of transplants and waitlist data, while SRTR delivers risk-adjusted, regression-based models to predict post-transplant outcomes. Currently, these models guide regulators, inform center evaluations, and shape policy decisions that profoundly affect patient care and resource allocation. These models, used to evaluate transplant center performance, are regression-based and often fall short in accuracy. SRTR C-statistics have been reported to range from 0.66 to 0.76 for kidney models, 0.67 to 0.83 for heart models,^2,18^ and 0.55 to 0.61 for lung models.^12^ Research-developed prediction scores also show room for improvement. Validated on 21,949 recipients from the UNOS database, the SOFT score achieves a C-statistic of 0.70.^19^ The BAR score achieves a C-statistic of 0.7, compared with 0.6 for D-MELD, which incorporates donor age into the MELD score.^20^ The Donor Risk Index and derived models have similar performance.^5^ Additional survival prediction scores following liver transplant have been evaluated, with several key models showing declining discrimination over time.^21^ Multiple scores have been developed for the prediction of kidney transplant outcomes,^22,23^ most notably the iBox system, which has been endorsed by the European Medicines Agency.^9^ The iBox prediction system predicted kidney allograft failure using 8 factors and was validated on 2129 European recipients (C-statistic 0.81, 95% confidence interval 0.78–0.84) and 1428 US recipients (0.80, 0.76–0.84).^9^ The LAS score predicts lung transplant outcomes with a C-statistic of 0.55 on UNOS data, and Chan et al.’s^24^ 2019 model performs similarly (C-statistic 0.59). For heart transplant, the IMPACT score achieved a C-statistic of 0.65 for 1-year survival on UNOS data.^8^ When tested on a smaller subset of 189 recipients from the Eurotransplant database, IMPACT predicted 3-month survival with a C-statistic of 0.76.^25^ Tested on 72 patients from a single center, IMPACT predicted in-hospital mortality with a C-statistic of 0.742.^26^ Remaining validations of the IMPACT score, including evaluation on UNOS data, report C-statistics below 0.65.^27^ The MELD score, widely used for waitlist prioritization, achieves a strong C-statistic of 0.83 for 3-month waitlist mortality.^28^ However, across liver, heart, lung, and kidney, no regression-based post-transplant prediction model has been validated on UNOS data with a C-statistic of 0.7 or greater.

The observed performance of XGBoost aligns with, and in important respects advances, a growing body of evidence supporting ML in transplantation. Prior ML models applied to transplant outcomes—including kidney,^9,10^ liver,^11^ lung,^12^ and heart^13,29–33^—have demonstrated promise but are largely constrained by small or single-center datasets lacking validation on the national UNOS registry. Four prior studies using UNOS data to predict post-transplant survival are most directly comparable to the present work. Kantidakis et al^14^ compared Cox regression to neural networks for post-transplant survival based on UNOS data and found that ML did not substantially outperform Cox, with integrated Brier scores of 0.180 for both model types. Guijo-Rubio et al^15^ similarly applied gradient boosting to UNOS liver transplant data across survival horizons of up to 9 years, achieving a clinically negligible difference in performance compared to 2 custom-tuned Cox proportional hazards models. Ershoff et al^16^ applied a deep neural network to a subset of UNOS data to predict 3-month post-transplant mortality, achieving an AUC of 0.703 compared with 0.655 and 0.688 by the SOFT and BAR scores, respectively—consistent with our liver model’s 1-year performance. Brahmbhatt et al^24^ similarly found that Least Absolute Shrinkage and Selection Operator and Random Forest models applied to UNOS lung transplant data failed to meaningfully outperform the LAS or other regression-based models for predicting 1-year post-transplant mortality, with AUCs of 0.55 to 0.62 across all models. Collectively, these studies suggest that ML gains over regression are modest when applied to pretransplant UNOS data, and none benchmarked their models directly against the SRTR regulatory Cox models specifically. The strongest prior performance was reported by Nitski et al.,^17^ who applied a Transformer deep learning architecture to longitudinal post-transplant laboratory data, achieving AUCs of 0.801 and 0.722 for 1-year and 5-year liver graft-survival, respectively. However, that approach relied on data accumulated after transplant—a richer information environment than the pretransplant prediction setting of the present study. Our results, demonstrating consistent XGBoost superiority over SRTR Cox models across all four organs using only pretransplant donor and recipient characteristics, represent the first direct head-to-head comparison of ML against the current regulatory models used to evaluate transplant center performance.

The results of this study build upon evidence that suggests ML may outperform regression in post-transplant predictive models. This is the first study to directly compare regression-based and ML-based methods using the same validating cohort employed in transplant regulatory models. More accurate regulatory models have the potential to enhance the regulation of centers, optimize outcomes, and reduce futile transplants. The mechanistic reasons for ML’s advantage likely stem from its inherent flexibility and adaptability. Unlike regression approaches, which impose linear or simple parametric relationships between predictors and outcomes, ML algorithms can model a wide array of complex interactions, including high-order nonlinearities and intricate variable interdependencies. For example, subtle donor-recipient characteristics or dynamic factors such as ischemic time may interact with immunologic parameters, comorbidities, and local center practices in ways that defy linear modeling. ML methods can leverage a greater number of predictors, identify latent patterns, and continuously evolve as more data become available. This is particularly salient in a field as heterogeneous as transplantation, where patient populations, donor characteristics, surgical techniques, and immunosuppressive protocols differ widely across centers and eras. Unlike regression, which offers limited scope for further refinement, ML continues to evolve, promising even greater accuracy as the field advances. The authors chose to measure prediction at 1 year and 3 years, given the clinical relevance of these time points, as clinicians often make decisions based on survival prediction at these horizons. Prospective validation is needed to confirm this study’s findings, but if ML continues to outperform regression, it could herald a paradigm shift in transplantation and all medical specialties.

The interpretability tradeoff between Cox regression and XGBoost represents a meaningful implementation barrier. While Cox models provide transparent hazard ratios that clinicians can directly inspect, XGBoost requires post hoc explainability tools such as SHAP (SHapley Additive exPlanations), which most transplant clinicians are not trained to interpret.^34^ More critically, opaque models risk embedding structural inequities from the UNOS registry—including disparities in access to care and racial biases—potentially disadvantaging centers serving marginalized communities. The current SRTR models are deliberately designed for program-level surveillance and prioritize fairness, transparency, and uncertainty quantification via confidence intervals; any ML replacement must satisfy these same regulatory objectives. Accordingly, Supplemental Tables 4–7, https://links.lww.com/AOSO/A653, provide SHAP-based feature importance (top 25 most important features per model), and Supplemental Figures 1–4, https://links.lww.com/AOSO/A653, provide representative beeswarm plots visualizing how feature values influence predictions for 1-year graft survival models. These analyses reveal that SRTR Cox model predictions themselves rank among the most important features (top 3–5 for nearly all organ-outcome pairs), indicating XGBoost leverages existing predictions as baseline signals while learning additional nonlinear patterns. Clinical factors (recipient age, dialysis duration for kidney, and life support for heart) dominate predictions, while demographic variables (race, insurance type) appear at lower ranks. Among the top 25 features across all 16 models, race/ethnicity variables appear in 8 models (ranks #11–#23) and insurance/payment variables appear in 7 models (ranks #4–#24). One kidney model (3-year graft survival) shows payment ranking as high as #4, though all other demographic variables rank outside the top 10 most important features. While demographic variables rank lower than clinical factors in most models, their presence among the top 25 features (17 occurrences across 400 total positions, 4.25%) warrants careful interpretation. Future deployment will require continued explainability analysis, clinician education on ML interpretability, uncertainty quantification via quantile regression or meta-bootstrapping, systematic subgroup analyses to ensure equitable predictions across demographic groups, and fairness-aware training algorithms to mitigate identified biases. These findings establish a proof-of-concept and underscore the need for further work balancing predictive accuracy with transparency and equity.

Several additional limitations must be acknowledged in this study. While the UNOS registry provides comprehensive data, our findings require prospective, external validation across diverse healthcare settings and international cohorts to confirm generalizability.^34^ All patient registries often suffer from inconsistencies and variability in data entry. Effort was taken to clean, standardize, and merge data, but persistent issues with data quality and completeness may limit the model’s predictive ability. In addition, though ML models excel in identifying subtle patterns, overfitting on training remains a risk. Although cross-validation and hyperparameter tuning were employed to minimize overfitting, ML models may still learn noise in the training data, potentially limiting their performance on new data. This concern is most salient for the lung cohort, where the improvement in 1-year graft AUC did not reach statistical significance—likely reflecting both the smaller lung transplant sample size relative to other organs and the comparatively high rate of early postoperative complications. Computationally, training XGBoost required moderate resources available on standard workstations (training time: minutes to hours per model; inference: near-instantaneous), without the use of specialized hardware. Regulatory deployment would require reproducible training pipelines, automated retraining workflows, and continuous monitoring infrastructure, demanding upfront engineering investment and collaboration among SRTR, UNOS, and Health Resources and Services Administration.

In conclusion, this study demonstrates that XGBoost models outperform SRTR Cox regression in predicting post-transplant survival across all 4 organs. By uncovering patterns and relationships beyond the scope of linear models, ML has the potential to inform a more refined and justifiable regulatory environment, improve patient care, and steer the field of transplantation toward more nuanced practices. Although ML models require post hoc explainability, bias auditing to prevent perpetuation of structural inequities, and prospective validation, these findings represent a significant stride forward. They suggest an impending transformation in transplantation toward the inclusion of ML models in predicting postoperative outcomes.

## Supplementary Material


