# Supplementary material for: XGBoost Outperforms Traditional Transplant Regulatory Models Based on Regression
Source: Ann Surg Open. 2026 Aug 5;7(3):e698. doi: 10.1097/AS9.0000000000000698 (PMC13596960; doi:10.1097/AS9.0000000000000698)

Supplemental Methods: The data preprocessing pipeline involved several critical steps to ensure data quality and model compatibility. Rather than imputing missing values, the native missing data handling capabilities of the modeling framework were leveraged, as tree-based ensemble methods can naturally accommodate missing values by learning optimal directions for missing data during the splitting process. However, features were systematically evaluated for excessive missingness patterns across class labels to identify and exclude variables where the presence or absence of data itself could indicate the outcome, thereby preventing spurious associations and data leakage. This involved calculating missingness rates stratified by outcome class and removing features where differential missingness exceeded predetermined thresholds, ensuring that the pattern of data availability did not become an inadvertent predictor. Categorical variables were transformed using one-hot encoding, creating binary indicator variables for each category level while avoiding multicollinearity through the removal of one reference category per variable. Feature engineering incorporated domain-specific knowledge from transplant medicine, including the creation of interaction terms between donor and recipient characteristics, temporal features capturing time-dependent effects, and composite scores reflecting organ quality and recipient acuity.

The model architecture was designed with careful consideration of the high-dimensional nature of transplant data and the need to capture complex nonlinear relationships between predictors and outcomes. The ensemble approach employed sequential model building where each successive model focused on correcting the errors of previous models, enabling the capture of intricate patterns in organ allocation and post-transplant outcomes. The maximum depth of individual components was constrained to prevent overfitting while maintaining sufficient complexity to model relevant interactions. Learning rate parameters controlled the contribution of each sequential component, balancing training efficiency with generalization performance. Regularization was implemented through constraints on model complexity, including limitations on the minimum number of observations required in terminal nodes and the maximum number of features considered at each split point.

Hyperparameter optimization was conducted through systematic exploration of the parameter space using cross-validation strategies tailored to the temporal nature of transplant data. The search process evaluated multiple hyperparameter combinations, including learning rates ranging from conservative to aggressive values, tree depth specifications from shallow to moderately deep structures, and various regularization strengths. The number of sequential components was determined through early stopping mechanisms that monitored validation set performance to identify the optimal point before overfitting occurred. Subsample ratios controlling the proportion of training data used for each component were varied to enhance model robustness and reduce overfitting. Column sampling parameters, which determine the fraction of features considered for each split, were tuned to introduce beneficial randomness and improve generalization. The optimization process employed nested cross-validation with stratification to ensure representative class distributions across folds, and performance was evaluated using clinically relevant metrics including discrimination, calibration, and decision curve analysis to assess net benefit across different threshold probabilities.

Supplemental Table 1: Demographics of sample, prior to data cleaning, stratified by organ transplanted.

Organ	Demographic	Value	Standard Deviation
Heart	Number of heart transplants	79,534	
	Number of heart transplants alone	78,252	
	Number of combined heart lung transplants	1,282	
	Age (years)	52.1	12.4
	Height (cm)	173.7	10.0
	Weight (kg)	80.4	17.5
	Male (%)	75.2%	
	BMI	26.6	4.9
	Underweight BMI	3.1%	
	Overweight BMI	35.6%	
	Class I obesity	18.4%	
	Class II obesity	4.7%	
	Class III obesity	1.4%	
	Life support	65.7%	
	Prior cardiac surgery	22.4%	
	Diagnosis - idiopathic dilated cariomyopathy	33.3%	
	Diagnosis - ischemic cardiomyopathy	28.1%	
	Diagnosis - coronary artery disease	11.2%	
Lung	Number of lung transplants	50,131	
	Number of lung transplants alone	48,849	
	Number of combined heart lung transplants	1,282	
	Age (years)	54.3	13.4
	Height (cm)	169.6	10.1
	Weight (kg)	72.3	16.9
	Male (%)	57.0	
	BMI	25.0	4.7
	Underweight BMI	9.1%	
	Overweight BMI	34.6%	
	Class I obesity	13.9%	
	Class II obesity	1.1%	
	Class III obesity	0.5%	
	Life support	10.2%	
	Disease group A (obstructive lung disease)	33.1%	

	Disease group B (pulmonary vascular disease)	6.2%	
	Disease group C (cystic fibrosis or immunodeficiency disorder)	9.9%	
	Disease group D (restrictive lung disease)	50.1%	
Liver	Number of liver transplants	186,873	
	Number of liver transplants alone	173,491	
	Number of combined liver kidney transplants	12,069	
	Age (years)	53.0	11.3
	Height (cm)	171.8	10.3
	Weight (kg)	83.1	19.8
	Male (%)	64.1%	
	BMI	28.1	5.8
	Underweight BMI	2.2%	
	Overweight BMI	33.9%	
	Class I obesity	20.2%	
	Class II obesity	8.9%	
	Class III obesity	4.2%	
	Life support	9.6%	
	Prior abdominal surgery	38.2%	
	Spontaneous bacterial peritonitis	7.3%	
	Portal vein thrombosis	4.7%	
	Transjugular intrahepatic portosystemic shunt	5.7%	
	Diagnosis - hepatitis C	23.7%	
	Diagnosis - alcoholic cirrhosis	20.6%	
	Diagnosis - non-alcoholic steatohepatitis	8.9%	
Kidney	Number of kidney transplants	559,156	
	Number of kidney transplants alone	532,192	
	Number of combined kidney pancreas transplants	26,964	
	Age (years)	49.0	13.8
	Height (cm)	170.4	10.7
	Weight (kg)	79.3	19.0
	Male (%)	60.6%	

	BMI	27.2	5.5
	Underweight BMI	2.7%	
	Overweight BMI	31.9%	
	Class I obesity	19.0%	
	Class II obesity	7.4%	
	Class III obesity	4.1%	
	Diagnosis - type I diabetes	7.2%	
	Diagnosis - type II diabetes	16.1%	
	Diagnosis - hypertensive nephropathy	16.2%	
	Diagnosis - polycystic kidney disease	6.8%	

Supplemental Table 2: Proportion of missing data for each dataset, stratified by era.

Organ	Dataset	Number of Features	Max Missing %	Min Missing %	Mean Missing %	Median Missing %	% Features with >50% Missing	% Features with 0% Missing
Heart	Full Dataset	1600	100	0	44.05	16.05	42.88	29.00
	2017-2019	1600	100	0	39.57	0.21	40.06	34.00
	2019-2022	1600	100	0	39.32	0.14	39.88	34.56
Lung	Full Dataset	1600	100	0	37.79	5.98	37.5	31.87
	2017-2019	1600	100	0	33.83	0.05	33.62	38.69
	2019-2022	1600	100	0	33.52	0.08	33.38	46.69
Liver	Full Dataset	1441	100	0	37.8	12.65	37.54	33.66
	2017-2019	1441	100	0	33.29	5.14	34.91	37.82
	2019-2022	1441	100	0	32.98	6.01	34.28	36.78
Kidney	Full Dataset	1390	100	0	40.95	26.3	43.38	33.45
	2017-2019	1390	100	0	37.27	29.65	36.55	36.47
	2019-2022	1390	100	0	36.28	24.81	35.83	35.47

Supplemental Table 3: Comparison of AUC values, Brier scores, sample sizes, cases, controls, and p-values for SRTR and XGBoost models across organ types and outcomes.

	Organ	Sample Size	Cases	Controls	SRTR Brier Score	XGBoost Brier Score	SRTR AUC	XGBoost AUC	AUC p-value
One-Year Graft	Heart	11,006	1,315	9,691	0.189	0.111	0.576	0.698	2.33 × 10^–7
	Lung	7,987	1,179	6,808	0.151	0.115	0.572	0.612	1.37 × 10^–1

Survival (2019–2022)	Liver	26,324	2,853	23,471	0.095	0.088	0.616	0.706	2.76 × 10^–6
	Kidney	67,788	5,306	62,482	0.072	0.064	0.649	0.736	4.70 × 10^–11
One-Year Patient Survival (2019–2022)	Heart	10,965	1,270	9,695	0.189	0.099	0.573	0.713	3.03 × 10^–4
	Lung	7,960	1,144	6,816	0.156	0.111	0.576	0.652	1.23 × 10^–2
	Liver	25,679	2,196	23,483	0.079	0.068	0.629	0.71	6.98 × 10^–5
	Kidney	66,385	3,330	63,055	0.048	0.048	0.656	0.763	6.06 × 10^–14
Three-Year Graft Survival (2017–2019)	Heart	9,012	1,452	7,560	0.265	0.123	0.555	0.674	2.27 × 10^–6
	Lung	7,015	2,120	4,895	0.212	0.207	0.558	0.614	3.18 × 10^–2
	Liver	21,177	3,522	17,655	0.139	0.135	0.583	0.621	3.23 × 10^–3
	Kidney	51,916	7,329	44,587	0.124	0.120	0.649	0.71	4.38 × 10^–14
Three-Year Patient Survival (2017–2019)	Heart	8,972	1,395	7,577	0.215	0.134	0.55	0.683	5.18 × 10^–6
	Lung	6,905	2,006	4,899	0.208	0.197	0.566	0.642	2.03 × 10^–2
	Liver	20,638	2,954	17,684	0.123	0.122	0.596	0.652	1.25 × 10^–4
	Kidney	50,473	5,046	45,427	0.092	0.107	0.688	0.764	1.75 × 10^–10

Supplemental Table 4: Top 25 most important features for XGBoost models of **Heart** transplants (indicated by SHAP analysis) and the proportion of each feature missing. (*Era evaluated for one-year models was 2019-2022 while era evaluated for three-year models was 2017-2019).

**Model**	**Feature**	**Mean |SHAP| (magnitude)**	**Missing % (Full Dataset)**	**Missing % (2019-2022)**
**One-Year Graft Survival (2019–2022)**	hr_reason_cd_ddr_heart_reason_code_502.0	0.14825699	0.27	0.32
	tbili_trr_total_bilirubin_mg_dl	0.13465287	17.05	0.34
	ischtime_ischemic_time_hours	0.12882032	3.14	0.34
	hr_reason_cd_ddr_heart_reason_code_501.0	0.1124195	0.27	0.32
	age_recipient_age	0.10854299	0.01	0.01
	creat_trr_trr_serum_creatinine_at_time_of_transplant	0.10564159	13.91	0.05
	prior_card_surg_tcr_tcr_prior_cardiac_surgery_non_transplant	0.09402215	7.45	13.23
	age_don_donor_age_yrs	0.09000026	0.01	0
	most_rcnt_creat_tcr_most_recent_creat.	0.05844761	30.13	0.24
	srtr_graft_survival_probability_3y	0.05356668	0	0
	vad_device_ty_trr_trr_patient_on_ventricular_assist_device_1.0	0.04924229	32.64	0
	dayswait_chron_total_days_on_waiting_list_including_inactive_time	0.04362159	0	0
	transfusions_trr_transfusions_since_listing	0.04042516	8.43	0

	init_age_wl_age_at_listing_in_years	0.03624435	0	0
	kdri_rao_kdri_rao_calculated_from_ddr_or_from_donornet_post_04_01_1994	0.03582742	15.32	0.05
	distance_distance_donor_hosp_to_tx_center_nautical_miles	0.03484567	1.38	0
	thoracic_dgn_wl_thoracic_diagnosis_1000.0	0.03449692	0	0
	hgt_cm_calc_calculated_recipient_height_cm	0.03325231	0.85	0
	days_stata4_days_in_adult_status_4	0.03203979	0	0
	region_region_5	0.03167374	0	0
	infect_iv_drug_trr_trr_infection_iv_drug_therapy_2_weeks_prior_to_tx	0.03118479	13.5	0.01
	hemo_sys_tcr_tcr_pa_sys_mm_hg	0.03105547	24.93	9.77
	hemo_pa_dia_trr_trr_pa_dia_mm_hg	0.02998541	24.18	8.75
	recipient_primary_diagnosis_qualifier_congenital_heart_disease	0.02991736	0	0
	func_stat_trr_trr_functional_status_transplant_2010.0	0.02965116	0.42	0.01
**One-Year Patient Survival (2019–2022)**	hr_reason_cd_ddr_heart_reason_code_502.0	0.17443673	0.27	0.32
	tbili_trr_total_bilirubin_mg_dl	0.13295837	17.05	0.34
	ischtime_ischemic_time_hours	0.13280827	3.14	0.34
	creat_trr_trr_serum_creatinine_at_time_of_transplant	0.10329171	13.91	0.05
	hr_reason_cd_ddr_heart_reason_code_501.0	0.09739031	0.27	0.32
	prior_card_surg_tcr_tcr_prior_cardiac_surgery_non_transplant	0.08855476	7.45	13.23
	age_recipient_age	0.07634762	0.01	0.01
	age_don_donor_age_yrs	0.06939352	0.01	0
	init_age_wl_age_at_listing_in_years	0.06380809	0	0
	most_rcnt_creat_tcr_most_recent_creat.	0.05871189	30.13	0.24
	srtr_graft_survival_probability_3y	0.05181877	0	0
	vad_device_ty_trr_trr_patient_on_ventricular_assist_device_1.0	0.04734692	32.64	0
	transfusions_trr_transfusions_since_listing	0.04686114	8.43	0
	region_region_5	0.04194844	0	0
	registration_to_admission_duration	0.04018787	26.11	0
	donor_s_organ_local_vs_shared_qualifier_local	0.03760219	0	0
	kdri_rao_kdri_rao_calculated_from_ddr_or_from_donornet_post_04_01_1994	0.03523982	15.32	0.05
	hemo_pa_dia_trr_trr_pa_dia_mm_hg	0.03499521	24.18	8.75
	thoracic_dgn_wl_thoracic_diagnosis_1000.0	0.03412375	0	0
	srtr_graft_survival_probability_1y	0.0331933	0	0
	distance_distance_donor_hosp_to_tx_center_nautical_miles	0.03224288	1.38	0

	func_stat_trr_trr_functional_status_transplant_2010.0	0.02860276	0.42	0.01
	infect_iv_drug_trr_trr_infection_iv_drug_therapy_2_weeks_prior_to_tx	0.02828881	13.5	0.01
	init_hgt_cm_calc_calculated_candidate_height_in_cm_at_listing	0.02803247	1.59	0.01
	hgt_cm_calc_calculated_recipient_height_cm	0.02790349	0.85	0
**Three-Year Graft Survival (2017–2019)**	hr_reason_cd_ddr_heart_reason_code_502.0	0.13201678	0.27	0.21
	hr_reason_cd_ddr_heart_reason_code_501.0	0.1063401	0.27	0.21
	ischtime_ischemic_time_hours	0.0955327	3.14	0.47
	days_stat1a_days_in_status_1a	0.09287095	0	0
	tbili_trr_total_bilirubin_mg_dl	0.08699884	17.05	0.12
	days_stata2_days_in_adult_status_2	0.08697254	0	0
	age_don_donor_age_yrs	0.07388094	0.01	0
	days_stata4_days_in_adult_status_4	0.06727243	0	0
	creat_trr_trr_serum_creatinine_at_time_of_transplant	0.06661806	13.91	0.03
	days_stat1b_days_in_status_1b	0.05980261	0	0
	prior_card_surg_tcr_tcr_prior_cardiac_surgery_non_transplant	0.05924399	7.45	14.11
	most_rcnt_creat_tcr_most_recent_creat.	0.0562143	30.13	0.22
	ethcat_ethnicity_category_2.0	0.05405024	0	0
	srtr_graft_survival_probability_1y	0.04227639	0	0
	cig_use_tcr_history_of_cigarette_use	0.03626533	40.33	0.01
	registration_to_admission_duration	0.03454825	26.11	0
	vad_device_ty_trr_trr_patient_on_ventricular_assist_device_1.0	0.03249397	32.64	0
	pri_payment_trr_trr_primary_source_of_payment_1.0	0.03112959	14.78	0
	age_recipient_age	0.03094224	0.01	0
	tcr_dgn_tcr_primary_diagnosis_at_listing_1000.0	0.03057217	0.18	0
	srtr_graft_survival_probability_3y	0.03013679	0	0
	dayswait_chron_total_days_on_waiting_list_including_inactive_time	0.02864534	0	0
	pri_payment_tcr_tcr_primary_projected_source_pay_1.0	0.02788525	14.78	0.01
	days_stata3_days_in_adult_status_3	0.02739998	0	0
	cmv_don_ddr_anti_cmv_serology_p_n_u_c_nd_i	0.02687035	0.2	0.05
**Three-Year Patient Survival**	hr_reason_cd_ddr_heart_reason_code_502.0	0.11419309	0.27	0.21
	days_stata2_days_in_adult_status_2	0.1023494	0	0
	ischtime_ischemic_time_hours	0.10078101	3.14	0.47
	creat_trr_trr_serum_creatinine_at_time_of_transplant	0.09757032	13.91	0.03

(2017–2019)	days_stat1a_days_in_status_1a	0.09280446	0	0
	age_don_donor_age_yrs	0.08557449	0.01	0
	days_stat1b_days_in_status_1b	0.08199033	0	0
	tbili_trr_total_bilirubin_mg_dl	0.07890583	17.05	0.12
	prior_card_surg_tcr_tcr_prior_cardiac_surgery_non_transplant	0.06320961	7.45	14.11
	hr_reason_cd_ddr_heart_reason_code_501.0	0.05970043	0.27	0.21
	ethcat_ethnicity_category_2.0	0.05043529	0	0
	days_stata4_days_in_adult_status_4	0.04872174	0	0
	srtr_graft_survival_probability_3y	0.047146	0	0
	pri_payment_trr_trr_primary_source_of_payment_1.0	0.04239325	14.78	0
	most_rcnt_creat_tcr_most_recent_creat.	0.0411025	30.13	0.22
	days_stata3_days_in_adult_status_3	0.04051631	0	0
	age_recipient_age	0.03932377	0.01	0
	init_age_wl_age_at_listing_in_years	0.03892867	0	0
	srtr_graft_survival_probability_1y	0.03772054	0	0
	cig_use_tcr_history_of_cigarette_use	0.03684343	40.33	0.01
	hematocrit_don_ddr_hematocrit	0.03680511	39.87	0.14
	pri_payment_tcr_tcr_primary_projected_source_pay_1.0	0.03640021	14.78	0.01
	dayswait_chron_total_days_on_waiting_list_including_inactive_time	0.03603693	0	0
	registration_to_admission_duration	0.03451472	26.11	0
	kdri_med_kdri_median_ref._donor_2022_median_calculated_from_ddr_or_from_donornet_post_04_01_1994	0.03083675	15.32	0.14

Supplemental Table 5: Top 25 most important features for XGBoost models of **Lung** transplants (indicated by SHAP analysis) and the proportion of each feature missing. (*Era evaluated for one-year models was 2019-2022 while era evaluated for three-year models was 2017-2019).

Model	Feature	Mean |SHAP| (magnitude)	Missing % (Full Dataset)	Missing % (Era*)
One-Year Graft Survival (2019–2022)	ischtime_ischemic_time_hours	0.11578871	4.07	0.38
	tbili_trr_total_bilirubin_mg_dl	0.08499043	6.09	0.13
	age_recipient_age	0.08409604	0	0
	lub_reason_cd_dr_double_lung_reason_code_502.0	0.07885242	34.71	22.21
	init_age_wl_age_at_listing_in_years	0.0717665	0	0
	srtr_graft_survival_probability_1y	0.05429159	0	0
	fvc_trr_trr_fvc	0.05102343	6.94	5.27

	recipient_medical_condition_qualifier_intensive_care	0.04751756	0	0
	init_creat_serum_creatinine_at_tcr_listing	0.0446527	15.8	0.06
	kdri_rao_kdri_rao_calculated_from_ddr_or_from_donornet_post_04_01_1994	0.04423295	4.9	0
	creatinine_current_creatinine	0.03756071	60.52	0.3
	ethcat_don_donor_ethnicity_category_1.0	0.03642605	0	0
	fev1_trr_trr_fev1	0.03241842	6.96	5.4
	six_min_walk_six_minute_walk_distance	0.03160664	27.69	0.88
	steroid_trr_chronic_steroid_use	0.03133235	3.75	0.09
	calc_six_min_walk_calculated_six_min_walk_distance	0.03090208	28.78	1.19
	srtr_graft_survival_probability_3y	0.03025359	0	0
	init_calc_las_initial_calculated_lung_allocation_score_pre_3_9_2023	0.02940635	28	0.01
	hbv_surf_total_trr_hbv_surface_antibody_total	0.02903991	59.69	4.49
	calc_creat_calculated_current_creatinine	0.02896612	60.54	0.34
	hemo_sys_trr_trr_pa_sys_mm_hg	0.0287056	14.75	4.49
	length_right_lung_length_of_right_lung_cm	0.02798539	51.77	11.47
	sodium170_val_don_ddr_serum_sodium_last_serum_sodium_prior_to_procurement	0.02790924	23.07	0
	transfusions_trr_transfusions_since_listing	0.02738916	1.22	0.09
	lt_lung_machine_perfusion_ddr_left_lung_machine_perfusion	0.02678902	59.47	2.33
**One-Year Patient Survival (2019–2022)**	age_recipient_age	0.14612076	0	0
	ischtime_ischemic_time_hours	0.10311893	4.07	0.38
	tbili_trr_total_bilirubin_mg_dl	0.07793714	6.09	0.13
	lub_reason_cd_dr_double_lung_reason_code_502.0	0.06885576	34.71	22.21
	srtr_graft_survival_probability_1y	0.05676274	0	0
	init_age_wl_age_at_listing_in_years	0.05537514	0	0
	init_creat_serum_creatinine_at_tcr_listing	0.05425592	15.8	0.06
	fvc_trr_trr_fvc	0.04501381	6.94	5.27
	recipient_medical_condition_qualifier_intensive_care	0.04331417	0	0
	six_min_walk_six_minute_walk_distance	0.04044657	27.69	0.88
	kdri_rao_kdri_rao_calculated_from_ddr_or_from_donornet_post_04_01_1994	0.03800264	4.9	0
	creatinine_current_creatinine	0.03483007	60.52	0.3
	init_calc_las_initial_calculated_lung_allocation_score_pre_3_9_2023	0.03413867	28	0.01
	steroid_trr_chronic_steroid_use	0.03360117	3.75	0.09
	hiv_don_ddr_hiv_don	0.03267032	0.3	0

	hbv_surf_total_trr_hbv_surface_antibody_total	0.02995862	59.69	4.49
	calc_bmi_calculated_bmi	0.02941258	25.95	0.04
	pco2_don_ddr_pco2	0.02940022	23.19	0.16
	bw6_candidate_most_recent_at_removal_bw6_antigen_from_waiting_list_95.0	0.02933303	0	0
	recipient_medical_condition_qualifier_not_hospitalized	0.02869179	0	0
	calc_six_min_walk_calculated_six_min_walk_distance	0.02835193	28.78	1.19
	rt_lung_machine_perfusion_ddr_right_lung_machine_perfusion	0.02748107	59.9	2.97
	ethcat_don_donor_ethnicity_category_1.0	0.02686606	0	0
	hemo_co_tcr_tcr_co_l_min	0.02667696	19.52	5.72
	srtr_graft_survival_probability_3y	0.0266721	0	0
**Three-Year Graft Survival (2017–2019)**	lub_reason_cd_dr_double_lung_reason_code_501.0	0.06952493	34.71	25.21
	init_age_wl_age_at_listing_in_years	0.04816933	0	0
	age_recipient_age	0.0474296	0	0
	init_creat_serum_creatinine_at_tcr_listing	0.04595174	15.8	0.12
	lub_reason_cd_dr_double_lung_reason_code_502.0	0.04501549	34.71	25.21
	dr51_2_candidate_most_recent_at_removal_dr51_antigen_from_waiting_list_0.0	0.03724325	0	0
	srtr_graft_survival_probability_3y	0.03679304	0	0
	kdri_rao_kdri_rao_calculated_from_ddr_or_from_donornet_post_04_01_1994	0.03590846	4.9	0.04
	six_min_walk_six_minute_walk_distance	0.03418877	27.69	0.94
	recipient_medical_condition_qualifier_not_hospitalized	0.03262137	0	0
	srtr_graft_survival_probability_1y	0.03079851	0	0
	ischtime_ischemic_time_hours	0.03049926	4.07	0.36
	ethcat_don_donor_ethnicity_category_1.0	0.0285698	0	0
	lub_disposition_dr_double_lung_disposition_6.0	0.02463396	34.63	25.16
	tbili_trr_total_bilirubin_mg_dl	0.02363656	6.09	0.13
	calc_six_min_walk_calculated_six_min_walk_distance	0.02147029	28.78	1.3
	cmv_don_ddr_anti_cmv_serology_p_n_u_c_nd_i	0.02042531	0.24	0.01
	recipient_medical_condition_qualifier_intensive_care	0.01917994	0	0
	dr53_2_candidate_most_recent_at_removal_dr53_antigen_from_waiting_list_0.0	0.01902729	0	0

	init_calc_las_initial_calculated_lung_allocation_score_pre_3_9_2023	0.01875527	28	0.03
	kdri_med_kdri_median_ref._donor_2022_median_calculated_from_ddr_or_from_donornet_post_04_01_1994	0.01872371	4.9	0.04
	recipient_transplant_procedure_type_qualifier_sequential	0.01767113	0	0
	iv_fluids_i.v._fluids_cc_hour	0.0166092	43.26	14.95
	fvc_trr_trr_fvc	0.01335682	6.94	1.68
	calc_fvc_abs_calculated_actual_fvc	0.01294618	29.73	2.71
**Three-Year Patient Survival (2017–2019)**	lub_reason_cd_dr_double_lung_reason_code_501.0	0.07881656	34.71	25.21
	lub_reason_cd_dr_double_lung_reason_code_502.0	0.07521063	34.71	25.21
	age_recipient_age	0.06170186	0	0
	init_age_wl_age_at_listing_in_years	0.05872655	0	0
	init_creat_serum_creatinine_at_tcr_listing	0.05072205	15.8	0.12
	six_min_walk_six_minute_walk_distance	0.05047578	27.69	0.94
	srtr_graft_survival_probability_3y	0.0477893	0	0
	lub_disposition_dr_double_lung_disposition_6.0	0.03630145	34.63	25.16
	kdri_rao_kdri_rao_calculated_from_ddr_or_from_donornet_post_04_01_1994	0.03472305	4.9	0.04
	ischtime_ischemic_time_hours	0.03297111	4.07	0.36
	recipient_medical_condition_qualifier_not_hospitalized	0.03150578	0	0
	dr51_2_candidate_most_recent_at_removal_dr51_antigen_from_waiting_list_0.0	0.02714522	0	0
	recipient_medical_condition_qualifier_intensive_care	0.02705797	0	0
	ethcat_don_donor_ethnicity_category_1.0	0.02351992	0	0
	srtr_graft_survival_probability_1y	0.02262599	0	0
	init_calc_las_initial_calculated_lung_allocation_score_pre_3_9_2023	0.02138337	28	0.03
	calc_six_min_walk_calculated_six_min_walk_distance	0.02119343	28.78	1.3
	tbili_trr_total_bilirubin_mg_dl	0.02065172	6.09	0.13
	cmv_don_ddr_anti_cmv_serology_p_n_u_c_nd_i	0.02051007	0.24	0.01
	dr53_2_candidate_most_recent_at_removal_dr53_antigen_from_waiting_list_0.0	0.02023733	0	0
	ebv_serostatus_trr_ebv_serostatus	0.01638699	13.09	0
	iv_fluids_i.v._fluids_cc_hour	0.01551965	43.26	14.95

	kdri_med_kdri_median_ref._donor_2022_median_calculated_from_ddr_or_from_donornet_post_04_01_1994	0.0154231	4.9	0.04
	pri_payment_tcr_tcr_primary_projected_source_pay_1.0	0.01497453	4.83	0
	end_blu_flg_lung_preference_at_removal_current_time_tcr_both_1_y	0.01335266	0	0

Supplemental Table 6: Top 25 most important features for XGBoost models of **Liver** transplants (indicated by SHAP analysis) and the proportion of each feature missing. (*Era evaluated for one-year models was 2019-2022 while era evaluated for three-year models was 2017-2019)

Model	Feature	Mean |SHAP| (magnitude)	Missing % (Full Dataset)	Missing % (Era*)
One-Year Graft Survival (2019–2022)	dcd_progressed_brain_death_if_no_was_this_an_authorized_dcd_donor_that_progressed_to_brain_death	0.10604822	49.25	15.53
	cold_isch_total_cold_ischemic_time_hours	0.09289213	5.03	0.7
	srtr_graft_survival_probability_1y	0.08194803	0	0
	inotrop_agents_don_ddr_greater_than_3_inotropic_agents_at_time_of_incision_y_n_pre_03_31_2015	0.07691645	37.28	100
	kdri_rao_kdri_rao_calculated_from_ddr_or_from_donornet_post_04_01_1994	0.07382149	13.91	6.01
	srtr_graft_survival_probability_3y	0.0733437	0	0
	exc_ever_was_an_exception_ever_submitted_for_pt_prior_6_28_2022	0.06100298	4.79	13.59
	age_recipient_age_yrs	0.04980903	0	0
	init_age_calculated_age_at_listing	0.04883337	0.81	0
	age_don_donor_age_yrs	0.04007769	0.03	0.01
	final_serum_sodium_wl_serum_sodium_at_removal	0.03963571	33.15	0.01
	li_reason_cd_dr_liver_reason_code_502.0	0.0358275	6.83	8.03
	final_serum_creat_wl_serum_creatinine_at_removal	0.03581674	25.23	0.06
	po2_to_fio2_ratio	0.03296163	34.03	6.36
	malig_trr_trr_any_known_malig._since_listing_pre_3_31_2015	0.03267974	34.76	100
	prev_tx_calculated_previous_liver_transplant	0.03170029	0	0
	func_stat_trr_trr_functional_status_transplant_2010.0	0.02987878	0.45	0.11

	dgn_tcr_tcr_primary_diagnosis_at_listing_4215.0	0.02950646	8.34	0
	on_vent_trr_trr_on_ventilator	0.02907631	0	0
	recipient_medical_condition_qualifier_not_hospitalized	0.02892402	0	0
	init_bilirubin_wl_bilirubin_at_listing	0.02885604	26.03	0.06
	hcv_serostatus_trr_hcv_serostatus	0.02767196	11.2	0.12
	portal_vein_trr_trr_risk_factor_portal_vein_thrombosis	0.02709941	0.04	0.1
	li_initial_flush_ddr_liver_initial_flush_solution_313.0	0.02667285	36.08	6.92
	kdri_med_kdri_median_ref._donor_2022_median_calculated_from_ddr_or_from_donornet_post_04_01_1994	0.02648618	13.91	6.01
**One-Year Patient Survival (2019–2022)**	inotrop_agents_don_ddr_greater_than_3_inotropic_agents_at_time_of_incision_y_n_pre_03_31_2015	0.09030625	37.28	100
	init_age_calculated_age_at_listing	0.09002075	0.81	0
	age_recipient_age_yrs	0.0809867	0	0
	cold_isch_total_cold_ischemic_time_hours	0.0801321	5.03	0.7
	hcv_serostatus_trr_hcv_serostatus	0.06723521	11.2	0.12
	exc_ever_was_an_exception_ever_submitted_for_pt_prior_6_28_2022	0.06100108	4.79	13.59
	srtr_patient_survival_probability_1y	0.06052226	0	0
	srtr_graft_survival_probability_1y	0.05401254	0	0
	dcd_progressed_brain_death_if_no_was_this_an_authorized_dcd_donor_that_progressed_to_brain_death	0.05350206	49.25	15.53
	li_reason_cd_dr_liver_reason_code_502.0	0.04917787	6.83	8.03
	heparin_heparin_y_n	0.04862833	63.07	27.15
	med_cond_trr_trr_medical_condition_3.0	0.04626546	0.04	0.04
	srtr_graft_survival_probability_3y	0.04428941	0	0
	albumin_tx_albumin_at_transplant	0.04339738	1.86	0
	final_serum_creat_wl_serum_creatinine_at_removal	0.04251171	25.23	0.06
	kdri_rao_kdri_rao_calculated_from_ddr_or_from_donornet_post_04_01_1994	0.04111212	13.91	6.01
	tbili_tx_total_bilirubin_transplant_mg_dl	0.03777704	0.57	0
	standardized_age	0.03765154	0	0
	init_serum_creat_wl_serum_creatinine_at_listing	0.03661264	26.37	0.14

	end_bmi_calc_calculated_candidate_bmi_at_removal_current_time	0.036354	1.78	0.03
	init_bilirubin_wl_bilirubin_at_listing	0.03550848	26.03	0.06
	creat_tx_serum_creatinine_at_time_of_transplant	0.03491311	0.66	0.06
	final_serum_sodium_wl_serum_sodium_at_removal_divided_by_init_serum_sodium_wl_serum_sodium_at_listing	0.03387664	35.29	0.08
	recipient_medical_condition_qualifier_not_hospitalized	0.03283817	0	0
	life_sup_trr_trr_patient_on_life_support	0.03195591	0.08	0.07
**Three-Year Graft Survival (2017–2019)**	li_reason_cd_dr_liver_reason_code_501.0	0.16152987	6.83	7.52
	li_reason_cd_dr_liver_reason_code_502.0	0.12183043	6.83	7.52
	dcd_progressed_brain_death_if_no_was_this_an_authorized_dcd_donor_that_progressed_to_brain_death	0.08757437	49.25	12.14
	share_ty_share_type_5.0	0.06884604	0	0
	hcv_serostatus_trr_hcv_serostatus	0.06346832	11.2	0
	cold_isch_total_cold_ischemic_time_hours	0.0624755	5.03	0.38
	kdri_rao_kdri_rao_calculated_from_ddr_or_from_donornet_post_04_01_1994	0.05752361	13.91	5.14
	srtr_graft_survival_probability_3y	0.0545537	0	0
	age_don_donor_age_yrs	0.05425933	0.03	0.01
	li_disposition_dr_liver_disposition_6.0	0.05405877	6.79	7.52
	init_age_calculated_age_at_listing	0.05154695	0.81	0
	srtr_graft_survival_probability_1y	0.0497288	0	0
	meld_diff_reason_cd_wl_reason_lab_meld_score_differs_from_match_meld_score_at_most_recent_time_excludes_active_exception_as_of_6_28_2022_3.0	0.04803243	71.53	63.43
	htlv_don_ddr_htlv_1_2_p_n_u_c_nd_i	0.04712902	12.85	5.14
	init_bilirubin_wl_bilirubin_at_listing	0.04084154	26.03	0.05
	prev_tx_calculated_previous_liver_transplant	0.04025661	0	0
	ddr52_donor_dr52_antigen_95.0	0.0348434	7.82	6.21
	recipient_medical_condition_qualifier_not_hospitalized	0.03433586	0	0
	li_alloc_stat_liver_allocation_score_or_status_at_transplant_post_6_28_2022	0.0321272	0.56	0
	init_inr_wl_inr_at_listing	0.03065492	26.04	0.05
	age_recipient_age_yrs	0.02987842	0	0
	albumin_tx_albumin_at_transplant	0.02966377	1.86	0
	life_sup_trr_trr_patient_on_life_support	0.0275357	0.08	0

	kdri_med_kdri_median_ref._donor_2022_median_calculated_from_ddr_or_from_donornet_post_04_01_1994	0.02668705	13.91	5.14
	creat_tx_serum_creatinine_at_time_of_transplant	0.02666997	0.66	0.04
**Three-Year Patient Survival (2017–2019)**	li_reason_cd_dr_liver_reason_code_501.0	0.13458534	6.83	7.52
	li_reason_cd_dr_liver_reason_code_502.0	0.10508078	6.83	7.52
	init_age_calculated_age_at_listing	0.07682531	0.81	0
	creat_tx_serum_creatinine_at_time_of_transplant	0.06433287	0.66	0.04
	heparin_heparin_y_n	0.06416295	63.07	23.51
	hcv_serostatus_trr_hcv_serostatus	0.06180449	11.2	0
	share_ty_share_type_5.0	0.06115657	0	0
	age_recipient_age_yrs	0.05602653	0	0
	recipient_medical_condition_qualifier_not_hospitalized	0.05251436	0	0
	init_bilirubin_wl_bilirubin_at_listing	0.05021533	26.03	0.05
	li_disposition_dr_liver_disposition_6.0	0.04766494	6.79	7.52
	cold_isch_total_cold_ischemic_time_hours	0.04677843	5.03	0.38
	srtr_graft_survival_probability_1y	0.04354746	0	0
	kdri_rao_kdri_rao_calculated_from_ddr_or_from_donornet_post_04_01_1994	0.04234792	13.91	5.14
	albumin_tx_albumin_at_transplant	0.0418518	1.86	0
	meld_diff_reason_cd_wl_reason_lab_meld_score_differs_from_match_meld_score_at_most_recent_time_excludes_active_exception_as_of_6_28_2022_3.0	0.03962795	71.53	63.43
	recipient_hcv_status_qualifier_not_positive	0.03597031	0	0
	dcd_progressed_brain_death_if_no_was_this_an_authorized_dcd_donor_that_progressed_to_brain_death	0.0359542	49.25	12.14
	li_alloc_stat_liver_allocation_score_or_status_at_transplant_post_6_28_2022	0.0353597	0.56	0
	registration_to_admission_duration	0.03455593	16.87	0
	age_don_donor_age_yrs	0.03362964	0.03	0.01
	srtr_graft_survival_probability_3y	0.03310016	0	0
	bun_don_ddr_terminal_blood_urea_nitrogen	0.02960529	13.02	5.14
	life_sup_trr_trr_patient_on_life_support	0.02952405	0.08	0
	init_wgt_kg_wl_tcr_candidate_weight_in_kg_at_listing_tcr	0.02810366	1.33	0

Supplemental Table 7: Top 25 most important features for XGBoost models of **Kidney** transplants (indicated by SHAP analysis) and the proportion of each feature missing. (*Era evaluated for one-year models was 2019-2022 while era evaluated for three-year models was 2017-2019).

Model	Feature	Mean |SHAP| (magnitude)	Missing % (Full Dataset)	Missing % (Era*)
**One-Year Graft Survival (2019–2022)**	age_recipient_age	0.09719949	0	0
	donor_circumstances_of_death_qualifier_suicide	0.09510174	26.12	24.81
	init_cpra_wl_current_pra_at_listing	0.08490392	47.99	0.17
	total_esrd_time_min_at_transplant	0.08236612	45.66	24.92
	srtr_patient_survival_probability_3y	0.07989606	0	0
	kir_reason_cd_dr_right_kidney_reason_code_501.0	0.07583851	27.81	26.24
	kil_reason_cd_dr_left_kidney_reason_code_501.0	0.07319138	27.81	26.23
	kdri_rao_kdri_rao_calculated_from_ddr_or_from_donornet_post_04_01_1994	0.07107317	36.07	24.81
	multiorgan_transplant_qualifier_missing	0.06878936	0	0
	num_org_tx_total_number_of_organs_transplanted	0.06613166	26.1	24.81
	tot_serum_album_tcr_total_serum_albumin	0.05514102	31.38	1.83
	end_cpra_candidate_most_recent_calculated_pra_up_to_offer_tx_removal_current_since_12_5_2007	0.05404037	41.74	0
	med_cond_trr_trr_medical_condition_pre_3_31_2015_3.0	0.05330435	35.89	100
	cold_isch_ki_kidney_cold_ischemic_time_hours	0.05177783	6.9	0.79
	pri_payment_tcr_ki_tcr_kidney_primary_projected_source_pay_1.0	0.0513403	12.17	0.04
	srtr_graft_survival_probability_3y	0.05062388	0	0
	work_income_trr_trr_working_for_income	0.04675409	33.58	3.19
	age_don_donor_age_yrs	0.04454092	0	0
	bun_don_ddr_terminal_blood_urea_nitrogen	0.04355308	35.41	24.81
	bw4_candidate_most_recent_at_removal_bw4_antigen_from_waiting_list_0.0	0.04296019	0	0
	kir_reason_cd_dr_right_kidney_reason_code_502.0	0.04193117	27.81	26.24
	wgt_kg_calc_calculated_recipient_weight_kg	0.04074911	1.06	0.02
	kil_reason_cd_dr_left_kidney_reason_code_502.0	0.0387588	27.81	26.23
	dial_trr_trr_dialysis_y_n	0.03780328	1.01	0.08
	inotrop_agents_inotrop_agents	0.03552299	52.15	100

**One-Year Patient Survival (2019–2022)**	age_recipient_age	0.17711924	0	0
	total_esrd_time_min_at_transplant	0.09122021	45.66	24.92
	candidate_diabetes_type_qualifier_none	0.08116693	0	0
	end_epts_offer_removal_current_calculated_epts_since_5_27_2014	0.07145303	63.88	3.12
	tot_serum_album_tcr_total_serum_albumin	0.06544973	31.38	1.83
	srtr_patient_survival_probability_3y	0.064579	0	0
	init_age_calculated_age_at_listing	0.0628008	0	0
	multiorgan_transplant_qualifier_missing	0.06267592	0	0
	recipient_age_at_transplant_qualifier_linear	0.06186711	0	0
	kil_reason_cd_dr_left_kidney_reason_code_501.0	0.06186032	27.81	26.23
	creat_trr_trr_serum_creatinine_at_time_of_transplant	0.05449279	19.7	0.93
	bw6_candidate_most_recent_at_removal_bw6_antigen_from_waiting_list_0.0	0.05312195	0	0
	dr51_2_candidate_most_recent_at_removal_dr51_antigen_from_waiting_list_0.0	0.0520706	0	0
	work_income_trr_trr_working_for_income	0.0513847	33.58	3.19
	srtr_patient_survival_probability_1y	0.05039877	0	0
	kir_reason_cd_dr_right_kidney_reason_code_501.0	0.04775599	27.81	26.24
	candidate_diabetes_status_type_at_onset_qualifier_none	0.04754305	0	0
	standardized_age	0.04615147	0	0
	pri_payment_tcr_ki_tcr_kidney_primary_projected_source_pay_1.0	0.04565232	12.17	0.04
	multiorgan_transplant_qualifier_no	0.04333528	0	0
	dial_trr_trr_dialysis_y_n	0.0426858	1.01	0.08
	end_cpra_candidate_most_recent_calculated_pra_up_to_offer_tx_removal_current_since_12_5_2007	0.04245132	41.74	0
	func_stat_trr_trr_functional_status_transplant_2090.0	0.03778384	0.05	0.08
	dr52_2_candidate_most_recent_at_removal_dr52_antigen_from_waiting_list_0.0	0.03743213	0	0
	med_cond_trr_trr_medical_condition_pre_3_31_2015_3.0	0.03633048	35.89	100
**Three-Year Graft Survival**	drugtrt_copd_tcr_drug_treated_copd_prior_3_31_2015_for_ki_kp	0.12971847	40.06	76.49
	donor_circumstances_of_death_qualifier_suicide	0.07290421	26.12	29.65
	num_org_tx_total_number_of_organs_transplanted	0.07269006	26.1	29.65

(2017–2019)	pri_payment_tcr_ki_tcr_kidney_primary_projected_source_pay_1.0	0.06676843	12.17	0
	kir_reason_cd_dr_right_kidney_reason_code_501.0	0.05969609	27.81	31.12
	age_recipient_age	0.05629539	0	0
	age_don_donor_age_yrs	0.04979877	0	0
	total_esrd_time_min_at_transplant	0.04926767	45.66	25.87
	cold_isch_ki_kidney_cold_ischemic_time_hours	0.04912618	6.9	0.62
	dr52_2_candidate_most_recent_at_removal_dr52_antigen_from_waiting_list_0.0	0.0465339	0	0
	dial_trr_trr_dialysis_y_n	0.04590647	1.01	0.01
	candidate_race_black_qualifier_yes	0.04580449	0	0
	ddr52_donor_dr52_antigen_95.0	0.04474213	28.03	29.68
	srtr_patient_survival_probability_3y	0.04251108	0	0
	end_cpra_candidate_most_recent_calculated_pra_up_to_offer_tx_removal_current_since_12_5_2007	0.04198849	41.74	0
	work_income_trr_trr_working_for_income	0.03842424	33.58	3.51
	creat_trr_trr_serum_creatinine_at_time_of_transplant	0.03788764	19.7	1.08
	peak_pra_wl_most_recent_peak_pra	0.03744868	55.92	100
	tot_serum_album_tcr_total_serum_albumin	0.03684345	31.38	2.23
	registration_to_admission_duration	0.03682928	18.64	0
	donor_smoking_history_qualifier_missing	0.03603834	0	0
	kil_reason_cd_dr_left_kidney_reason_code_501.0	0.03592578	27.81	31.12
	hiv_don_ddr_hiv_don	0.03456525	26.16	29.65
	kdri_med_kdri_median_ref._donor_2022_median_calculated_from_ddr_or_from_donornet_post_04_01_1994	0.03404347	36.07	29.65
	diag_ki_kidney_diagnosis_from_trr_tcr_3008.0	0.03323913	1.32	0.53
**Three-Year Patient Survival (2017–2019)**	age_recipient_age	0.36579904	0	0
	drugtrt_copd_tcr_drug_treated_copd_prior_3_31_2015_for_ki_kp	0.18867068	40.06	76.49
	candidate_diabetes_type_qualifier_none	0.14458728	0	0
	srtr_patient_survival_probability_3y	0.09615231	0	0
	total_esrd_time_min_at_transplant	0.09381129	45.66	25.87
	creat_trr_trr_serum_creatinine_at_time_of_transplant	0.09175332	19.7	1.08
	work_income_trr_trr_working_for_income	0.06900197	33.58	3.51
	registration_to_admission_duration	0.05918565	18.64	0
	med_cond_trr_trr_medical_condition_pre_3_31_2015_3.0	0.0572094	35.89	100

	multiorgan_transplant_qualifier_missing	0.05571972	0	0
	dr52_2_candidate_most_recent_at_removal_dr52_antigen_from_waiting_list_0.0	0.04911848	0	0
	pri_payment_tcr_ki_tcr_kidney_primary_projected_source_pay_1.0	0.04697746	12.17	0
	dayswait_alloc_time_used_for_allocation_priority_days	0.04688061	21.57	1.43
	dial_trr_trr_dialysis_y_n	0.04688017	1.01	0.01
	ddr52_donor_dr52_antigen_95.0	0.04605559	28.03	29.68
	peak_pra_wl_most_recent_peak_pra	0.04604526	55.92	100
	num_org_tx_total_number_of_organs_transplanted	0.045754	26.1	29.65
	cold_isch_ki_kidney_cold_ischemic_time_hours	0.04541138	6.9	0.62
	ethnicity_tcr_ethnicity	0.0448555	0	0
	prev_preg_trr_previous_pregnancies	0.04442158	60.85	60.72
	tot_serum_album_tcr_total_serum_albumin	0.04405914	31.38	2.23
	func_stat_trr_trr_functional_status_transplant_2090.0	0.04079122	0.05	0.01
	hiv_don_ddr_hiv_don	0.03887487	26.16	29.65
	bun_don_ddr_terminal_blood_urea_nitrogen	0.03786503	35.41	29.65
	kil_reason_cd_dr_left_kidney_reason_code_501.0	0.03781897	27.81	31.12

Supplemental Figures 1-4: Beeswarm plots produced by Shapley Additive exPlainations (SHAP) analysis. In each figure, each point represents a patient, with color indicating feature value (red = high, blue = low) and horizontal position showing contribution to risk prediction.

Supplemental Figure 1: Top 25 Features for XGBoost Heart 1-year Graft Survival (Beeswarm plot).

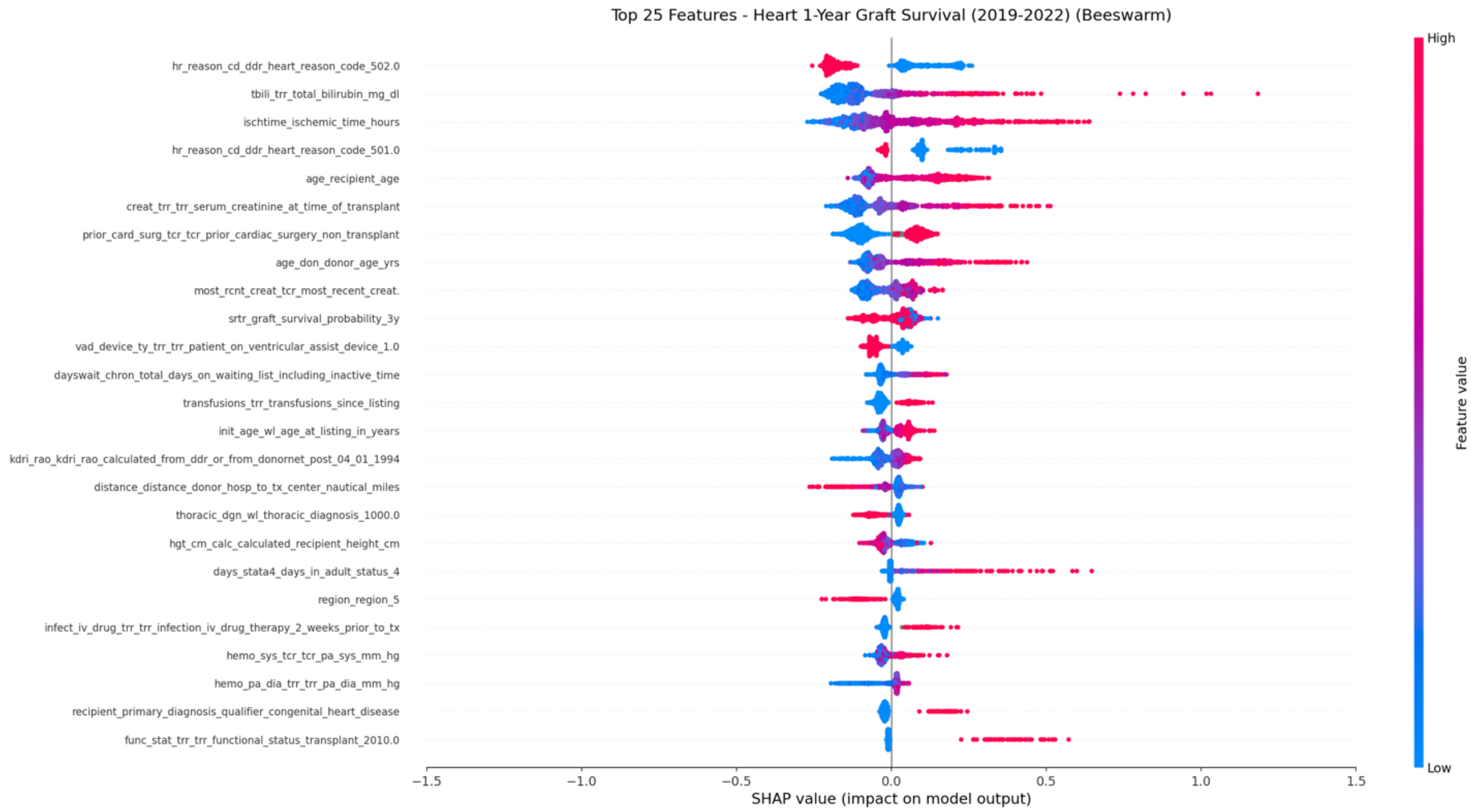

Supplemental Figure 2: Top 25 Features for XGBoost Lung 1-year Graft Survival (Beeswarm plot).

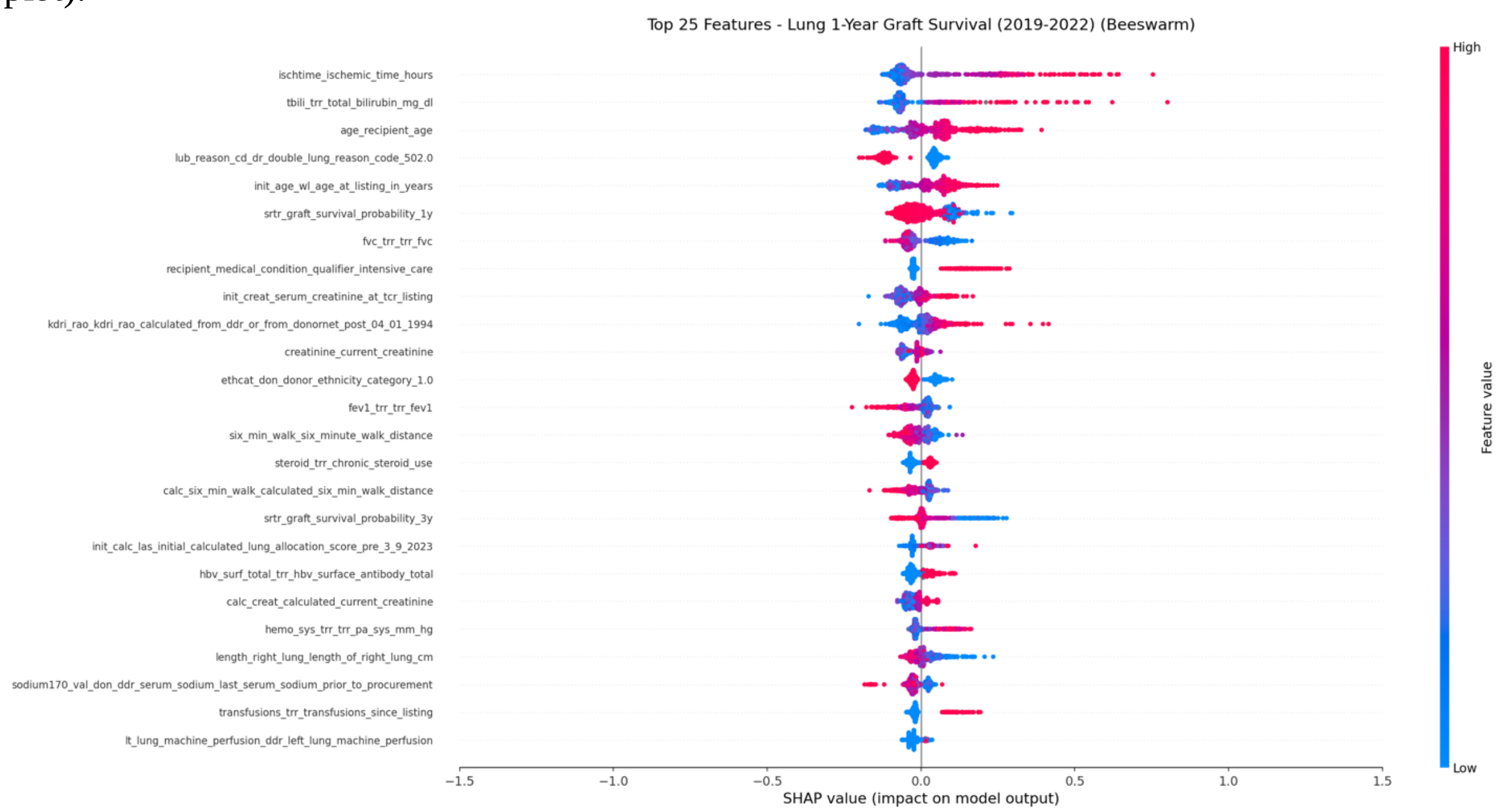

Supplemental Figure 3: Top 25 Features for XGBoost Liver 1-year Graft Survival (Beeswarm plot).

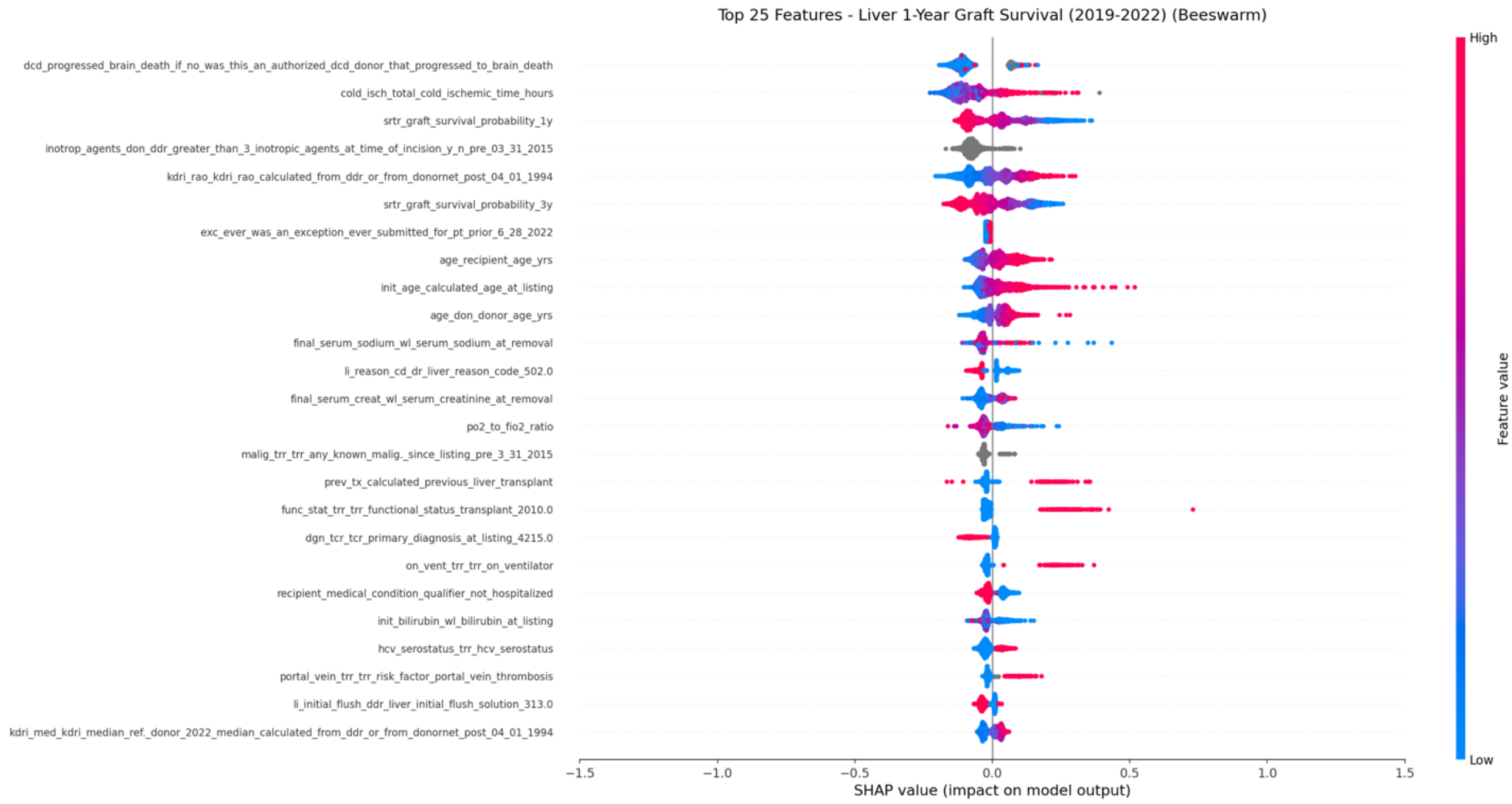

Supplemental Figure 4: Top 25 Features for XGBoost Kidney 1-year Graft Survival (Beeswarm plot).

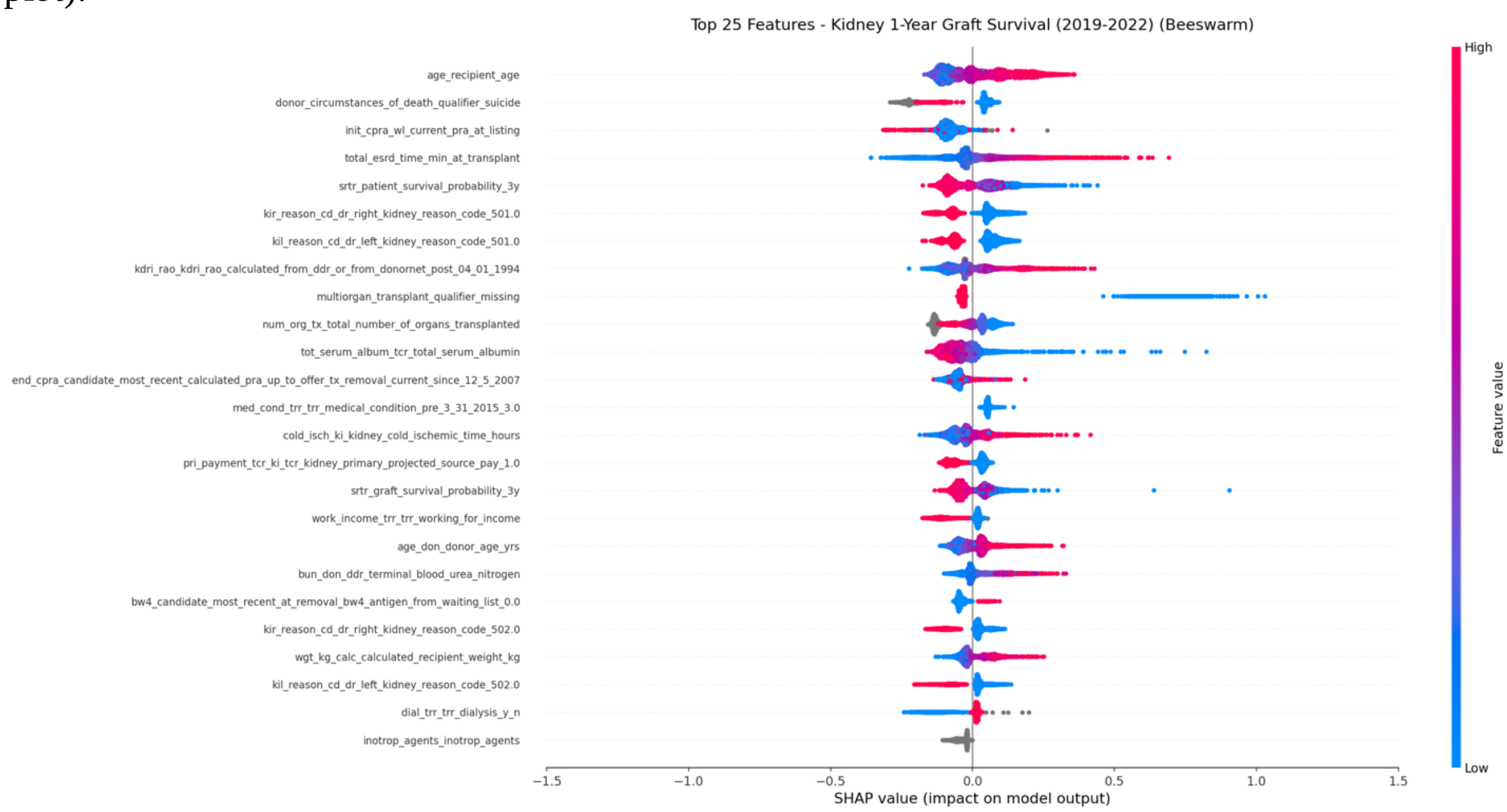

Supplement: Supplementary file 1 [file as9-7-e698-s001.pdf]
